# CAMLG-CDG: a novel congenital disorder of glycosylation linked to defective membrane trafficking

**DOI:** 10.1093/hmg/ddac055

**Published:** 2022-03-09

**Authors:** Matthew P Wilson, Zoé Durin, Özlem Unal, Bobby G Ng, Thomas Marrecau, Liesbeth Keldermans, Erika Souche, Daisy Rymen, Mehmet Gündüz, Gülşen Köse, Luisa Sturiale, Domenico Garozzo, Hudson H Freeze, Jaak Jaeken, François Foulquier, Gert Matthijs

**Affiliations:** Laboratory for Molecular Diagnosis, Center for Human Genetics, KU Leuven, Leuven 3000, Belgium; CNRS, UMR 8576 - UGSF Unité de Glycobiologie Structurale Et Fonctionnelle, University of Lille, Villeneuve D'Ascq, 59000, Lille, France; Division of Pediatric Metabolism and Nutrition, Ankara Children’s Training and Research Hospital, Ankara, Turkey; Human Genetics Program, Sanford Burnham Prebys Medical Discovery Institute, La Jolla, CA 92037, USA; Laboratory for Molecular Diagnosis, Center for Human Genetics, KU Leuven, Leuven 3000, Belgium; Laboratory for Molecular Diagnosis, Center for Human Genetics, KU Leuven, Leuven 3000, Belgium; Laboratory for Molecular Diagnosis, Center for Human Genetics, KU Leuven, Leuven 3000, Belgium; Department of Pediatrics, Center for Metabolic Diseases, University Hospitals Leuven, Leuven 3000, Belgium; Division of Pediatric Metabolism and Nutrition, Ankara Children’s Training and Research Hospital, Ankara, Turkey; Department of Pediatric Neurology, Şişli Etfal Education and Research Hospital, Istanbul, Turkey; CNR, Institute for Polymers, Composites and Biomaterials, IPCB, Catania, Italy; CNR, Institute for Polymers, Composites and Biomaterials, IPCB, Catania, Italy; Human Genetics Program, Sanford Burnham Prebys Medical Discovery Institute, La Jolla, CA 92037, USA; Department of Pediatrics, Center for Metabolic Diseases, University Hospitals Leuven, Leuven 3000, Belgium; CNRS, UMR 8576 - UGSF Unité de Glycobiologie Structurale Et Fonctionnelle, University of Lille, Villeneuve D'Ascq, 59000, Lille, France; Laboratory for Molecular Diagnosis, Center for Human Genetics, KU Leuven, Leuven 3000, Belgium

## Abstract

The transmembrane domain recognition complex (TRC) pathway is required for the insertion of C-terminal tail-anchored (TA) proteins into the lipid bilayer of specific intracellular organelles such as the endoplasmic reticulum (ER) membrane. In order to facilitate correct insertion, the recognition complex (consisting of BAG6, GET4 and UBL4A) must first bind to TA proteins and then to GET3 (TRC40, ASNA1), which chaperones the protein to the ER membrane. Subsequently, GET1 (WRB) and CAML form a receptor that enables integration of the TA protein within the lipid bilayer. We report an individual with the homozygous c.633 + 4A>G splice variant in *CAMLG*, encoding CAML. This variant leads to aberrant splicing and lack of functional protein in patient-derived fibroblasts. The patient displays a predominantly neurological phenotype with psychomotor disability, hypotonia, epilepsy and structural brain abnormalities. Biochemically, a combined O-linked and type II N-linked glycosylation defect was found. Mislocalization of syntaxin-5 in patient fibroblasts and in si*CAMLG* deleted Hela cells confirms this as a consistent cellular marker of TRC dysfunction. Interestingly, the level of the v-SNARE Bet1L is also drastically reduced in both of these models, indicating a fundamental role of the TRC complex in the assembly of Golgi SNARE complexes. It also points towards a possible mechanism behind the hyposialylation of *N* and *O*-glycans. This is the first reported patient with pathogenic variants in *CAMLG*. CAMLG-CDG is the third disorder, after GET4 and GET3 deficiencies, caused by pathogenic variants in a member of the TRC pathway, further expanding this novel group of disorders.

## Introduction

**Figure 1:**
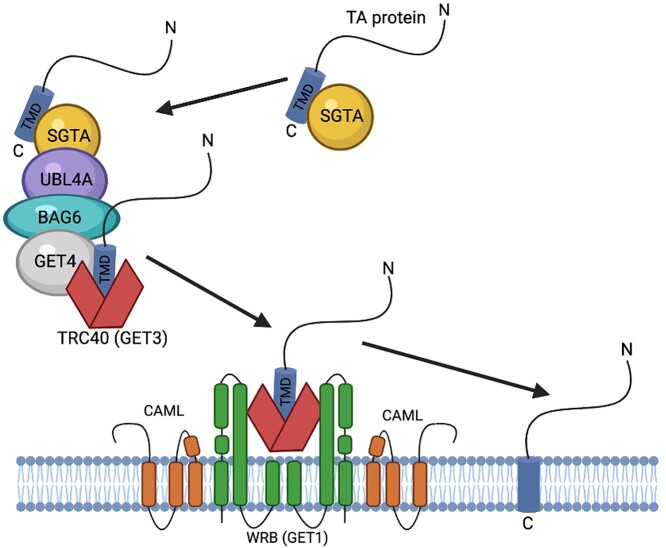
The mammalian TRC pathway.

A significant proportion of human proteins are membrane-bound. For plasma membrane proteins, the canonical pathway is that of the secretory pathway via the endoplasmic reticulum (ER) and Golgi. However, a subset of membrane-bound proteins follow less conventional pathways. Among these are the cytoplasmically facing tail-anchored (TA) proteins that have a single hydrophobic transmembrane domain (TMD) at their C-terminus and therefore must be post-translationally inserted into the lipid bilayer of the ER, peroxisome or mitochondria (1,2). For TA proteins targeted to the ER, this process is performed, in mammals, by the transmembrane domain recognition complex (TRC) pathway that consists first of a recognition complex (composed of BAG6, GET4 and UBL4A), which binds to TA proteins and then to GET3 (TRC40, ASNA1) (3–5). GET3 chaperones the TA protein to the ER membrane where GET1 (WRB) and CAML form a receptor which enables its integration within the lipid bilayer (Fig. 1) (6,7). This pathway is conserved in all eukaryotes, although the CAML subunit of the TRC receptor has several homologs in different organisms (3).

Many ER-targeted TA proteins are involved in the vesicular targeting and fusion mechanisms and hence in the conventional trafficking of proteins between the Golgi and ER. Among these are many members (e.g. STX5, STX6, VAMP7, SEC22B, VTI1A) or interactors (e.g. VAPA, VAPB) of SNAREs, as well as some proteins that are later trafficked to the nuclear membrane (e.g. CUX1, EMD) (8).

Recently, pathogenic compound heterozygous missense variants (c.365G>A; p.(Arg122His), c.837A>G; p.(Ile279Met)) in *GET4* were reported in an individual with intellectual disability (ID), seizures, facial dysmorphism and some skeletal abnormalities (9). The affected individual had a mild type 2 serum transferrin isoform pattern, indicating a possible congenital disorder of glycosylation (CDG) caused by dysfunction of the TRC pathway. In addition, in 2019 an individual with compound heterozygous variants (c.913C>T; p.(Gln305^*^), c.488T>C; p.(Val163Ala)) in *GET3* was described (10). This patient presented with severe neonatal-onset cardiomyopathy leading to death at age seven weeks. Biochemically, abnormal serum transferrin and apolipoprotein C-III pointed to a combined N- and O-linked glycosylation defect.

In this study, we describe a patient with the homozygous splice variant c.633 + 4A>G in *CAMLG* (chr5:134741527A>G (GRCh38.p13))*,* the gene encoding CAML, an essential subunit of the TRC pathway receptor. The patient presented with a mostly neurological disorder characterized by severe developmental delay (DD) and ID, hypotonia, limb contractures, seizures and structural brain abnormalities as well as combined serum N- and O-linked glycosylation defects, confirming TRC pathway defects as congenital disorders of glycosylation.

## Results

### Clinical summary

The affected male was born at term in 2007 with birth weight of 2500 g and head circumference of 35 cm. He is the second child of consanguineous Turkish parents (fourth degree cousins). The first child was healthy and there is no family history of metabolic diseases.

The family noticed developmental delay at three months of age, since he had no head control. Smiling and eye contact were appropriate for his age, but head control was never achieved. He was babbling at one year, but seizures started at that age and he lost his milestones. Seizures consisted of staring at a point accompanied by stiffness and unresponsiveness. He has been seizure free the last 4 years since receiving levetiracetam treatment. He can only sit with considerable support and has limb contractures. He has a gastrostomy and has been receiving intermittent respiratory support for 6 months as of publication.

Brain MRI at 7 years showed a very thin corpus callosum, atrophic brain stem, severe cerebral and cerebellar atrophy and diffuse hypomyelination.

**Figure 2:**
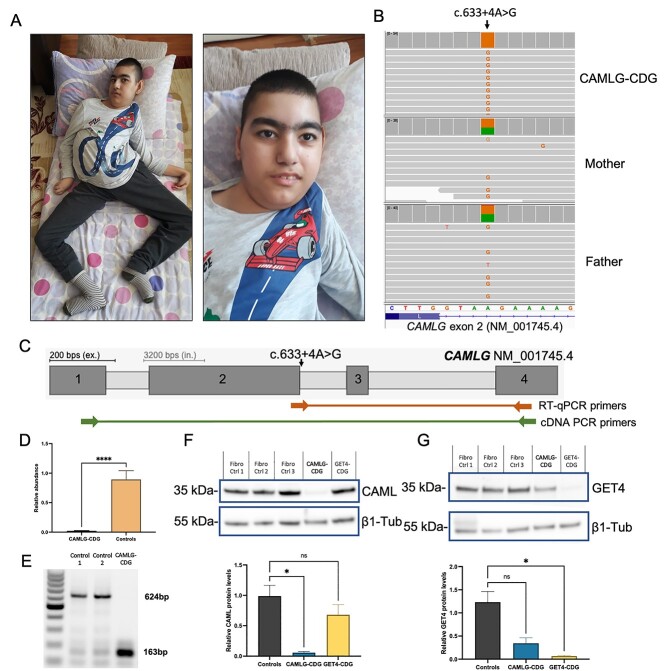
The c.633 + 4A>G variant in *CAMLG* leads to skipping of exon 2 and absence of the CAML protein in affected fibroblasts. (**A**) Photographs of the patient aged 14 years. (**B**) The homozygous c.633 + 4A>G variant in *CAMLG* (NM_001745.4; chr5:134741527A>G (GRCh38.p13)), inherited in an autosomal recessive fashion. (**C**) Schematic of *CAMLG* and the primers used for RT-qPCR and PCR amplification of cDNA made from fibroblast *CAMLG* mRNA. (**D**) RT-qPCR shows a reduction in levels of the canonical *CAMLG* transcript in cDNA made from mRNA from patient and control fibroblasts. (**E**) Gel electrophoresis of amplicons produced by the cDNA PCR primers indicated in C show skipping of exon 2 of *CAMLG* in cDNA transcribed from RNA in patient fibroblasts. (**F**) Steady state protein levels of CAML are reduced in CAMLG-CDG patient fibroblasts, but unaffected in GET4 deficiency fibroblasts. (**G**) Steady state protein levels of GET4 are reduced in GET4 deficiency patient fibroblasts, and lowered in CAMLG-CDG fibroblasts (ns; *P* = 0.054). Error bars = SEM. NS = not significant (*P* < 0.05); ^^*^^ = *P* < 0.05; ^^*^^*^^*^^*^ ^= *P* < 0.0001.

The most recent follow-up at 14 years showed weight of 30.6 kg (below 3rd centile), height could not be measured due to contractures, and head circumference of 46 cm (below 3rd centile). He has a dolichocephalic head but no other significant dysmorphism (Fig. 2A). He has axial hypotonia, limb spasticity and contractures. He has contact with his environment but makes only sounds. Eye movements are normal. Deep tendon reflexes are obtained, plantar reflexes are flexor, but he has a bilateral clonus. There is no organomegaly.

Routine blood count and laboratory parameters are normal. Serum transferrin isoelectrofocusing (IEF) repeatedly showed a type 2 pattern.

Genetic analysis using a panel of known ‘CDG genes’ did not reveal any potentially pathogenic variants. Therefore, trio whole genome analysis was performed and identified the c.633 + 4A>G variant in *CAMLG*, inherited in an autosomal recessive fashion (Fig. 2B).

*In silico* prediction tools suggest the c.633 + 4A>G variant in *CAMLG* leads to skipping of exon 2 of the canonical *CAMLG* transcript (NM_001745.4). In order to confirm this *in vivo*, primers spanning the exon–exon boundary between exons 2 and 3 were designed (Fig. 2C; RT-qPCR primers). Indeed, on analysis of mRNA from patient fibroblasts, expression of the canonical *CAMLG* transcript was negligible (Fig. 2D). Furthermore, PCR products created by amplification of cDNA using primers stretching from exon 1 to exon 4 of *CAMLG* (Fig. 2C; cDNA PCR primers) were visualized by gel electrophoresis. This showed that the canonical full-length amplicon of 624 bp (containing parts of all 4 exons) was absent in affected fibroblasts. In addition, a corresponding increase of a truncated ~163 bp amplicon could be seen in affected fibroblasts, confirming that the c.633 + 4A>G variant causes skipping of exon 2 (containing 461 nucleotides and the codons for 154 amino acid codons) and the fusion of exons 1 and 3 (Fig. 2E). On a protein level, this is predicted to result in a truncated protein product p.(Glu58ValfsTer80).

Accordingly, reduced levels of CAML protein were identified using immunoblotting analysis (*P* < 0.05) (Fig. 2F). In fibroblasts from the previously identified individual with pathogenic variants in *GET4*, CAML steady state levels were normal. Intriguingly, GET4 levels seemed to also be decreased compared to controls (71% reduction), although this change was not significant and not as strong as the reduction seen in the individual with GET4 deficiency (Fig. 2G).

**Figure 3:**
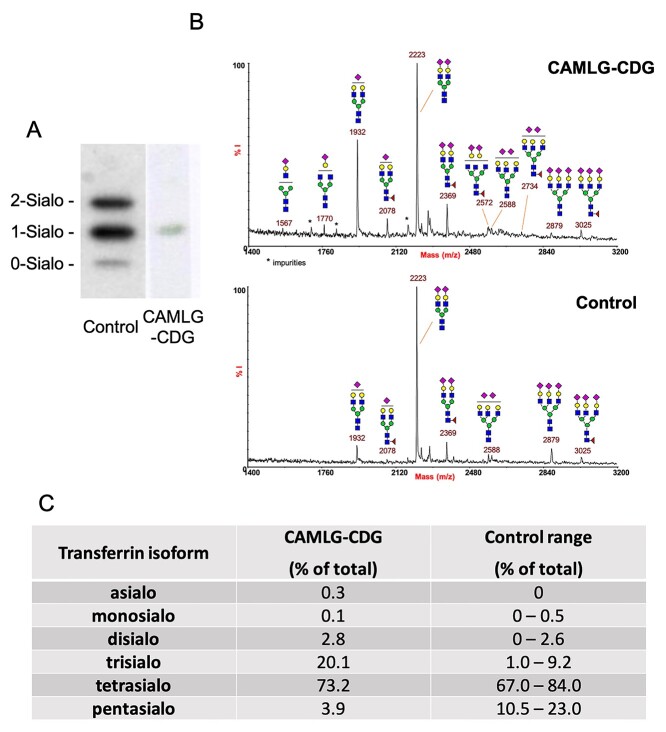
Serum glycosylation abnormalities show combined type II N-linked and O-linked glycosylation defects. (**A**) ApoC-III isoelectric focusing profile showing an abnormal pattern in the affected CAML deficient individual, with absent apoC-III_2_ and apoC-III_0_, and the presence of only small amounts of apoC-III_1_. (**B**) MALDI-TOF mass spectra (linear mode and negative polarity) of acidic *N*-glycans from serum Tf showing a CDG-II pattern. (**C**) Quantification of transferrin glycoforms measured by capillary zone electrophoresis showing reduced pentasialo transferrin and raised trisialo transferrin.

Isoelectric focusing analysis of serum apoC-III glycoforms showed an absence of asialo and disialo apoC-III, and only a small amount of monosialo apoC-III (Fig. 3A). This indicates a defect of mucin type O-glycosylation (11). Serum transferrin glycoforms analyzed by matrix-assisted laser desorption—ionization-time of flight (MALDI-TOF) mass spectrometry and capillary zone electrophoresis (CZE) also indicated a type II N-glycosylation defect (Fig. 3B and C). The clear combined O- and N-linked glycosylation defects in serum points to general glycosylation abnormalities associated with TRC pathway dysfunction. The observed lack of sialylation on both O- and N-linked glycan on serum glycoproteins argues for an impact of CAMLG on late Golgi function.

**Figure 4:**
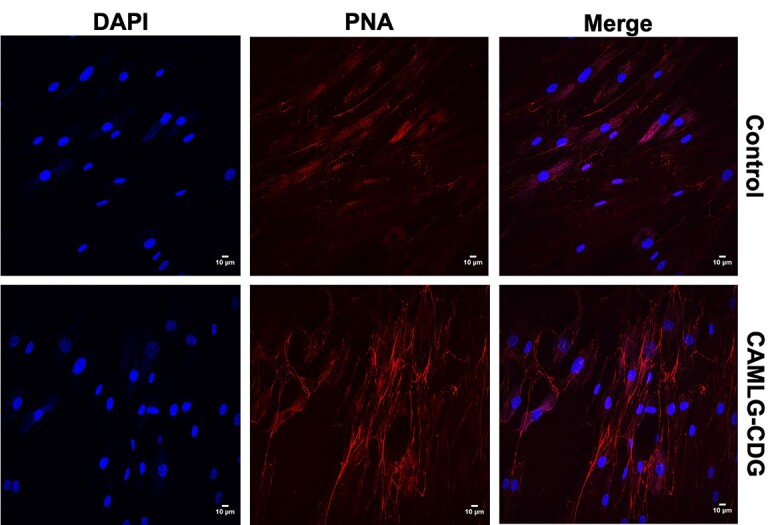
Increased peanut agglutinin (PNA) staining in affected CAMLG-CDG fibroblasts indicate an O-glycan sialylation defect. PNA binds to O-glycans containing terminal Galß1,3GalNAc. Addition of terminal sialic acid to Gal prevents lectin binding. The large increased signal in affected CAMLG-CDG fibroblasts shows that sialylation is deficient in these cells.

To confirm the glycosylation abnormalities found in liver-derived serum proteins transferrin and apoC-III, we stained fibroblasts with the peanut agglutinin (PNA) lectin that recognizes the asialylated carbohydrate sequence galactose- β(1-3)-*N*-acetylgalactosamine (Gal-β(1-3)-GalNAc). An elevated level of PNA binding indicates hyposialylation of O-glycans, which is seen in other CDG such as deficiencies of the conserved oligomeric Golgi (COG) complex as a signature of general Golgi dysfunction (12). Indeed, although PNA lectin fluorescence was barely detectable in control fibroblasts, a strong increase in signal was identified in affected fibroblasts (Fig. 4A). Interestingly, staining with *vicia villosa* lectin (VVL), which recognizes α- or β-linked terminal *N*-acetylgalactosamine, showed no increased signal in affected fibroblasts (Supplementary Material, Fig. S1). This demonstrates that the defect only affects the sialylation of core 1 *O*-glycans rather than the initiation process with the transfer of GalNAc and galactose. Fibroblast markers of abnormal N-linked glycosylation previously used (LAMP1, LAMP2, ICAM-1; (13)) were tested in order to identify hypoglycosylation but no abnormalities could be identified (data not shown).

It was hypothesized that the combined N- and O-linked sialylation defect could be due to aberrant processing or localization of sialyltransferases within the late Golgi. In order to test this, HeLa cells stably expressing green fluorescent protein (GFP)-tagged β-galactoside alpha-2,6-sialyltransferase 1 (ST6GAL1) were subjected to siRNA-based knockdown of *CAMLG*, resulting in only ±2% residual CAML protein after siRNA treatment (Supplementary Material, Fig. S2). Treated cells showed normal levels of ST6GAL1, but with a more fragmented distribution (Supplementary Material, Fig. S3). This indicated that knockdown of *CAMLG* expression leads to at least some disorganization of the Golgi.

Previously, it was shown that both the long and short forms of syntaxin-5 (STX) were mislocalized to the cytoplasm in fibroblasts from an individual with pathogenic compound heterozygous variants in *GET4* (9). The same was also true for the present variant in *CAMLG*. Crude fractionation of affected CAMLG-CDG fibroblasts showed a significant increase in the amount of STX5 mislocalized to the cytoplasm. This was identified in both the long (*P* < 0.05) and short (*P* < 0.001) forms of STX5 and also found in parallel in GET4 deficient fibroblasts (9) (Supplementary Material, Fig. 5A). Total STX5 steady state protein levels were normal in both CAMLG-CDG and GET4 deficient fibroblasts (Supplementary Material, Fig. S4). Interestingly, a larger proportion of the short form of STX5 (STXS; CAMLG-CDG = 43%; GET4-CDG 50%) appeared to be mislocalized to the cytoplasm than the long form (STX5L; CAMLG-CDG = 9%; GET4-CDG = 20%). The mislocalization of STX5 was also visualized by immunofluorescence staining, with significantly lower amounts of STX5 colocalizing with the Golgi marker giantin (Fig. 6A; Supplementary Material, Fig. S5; Pearsons *R*: *P* < 0.0001). This data confirm that STX5 mislocalization is a cellular marker of TRC pathway dysfunction.

**Figure 5:**
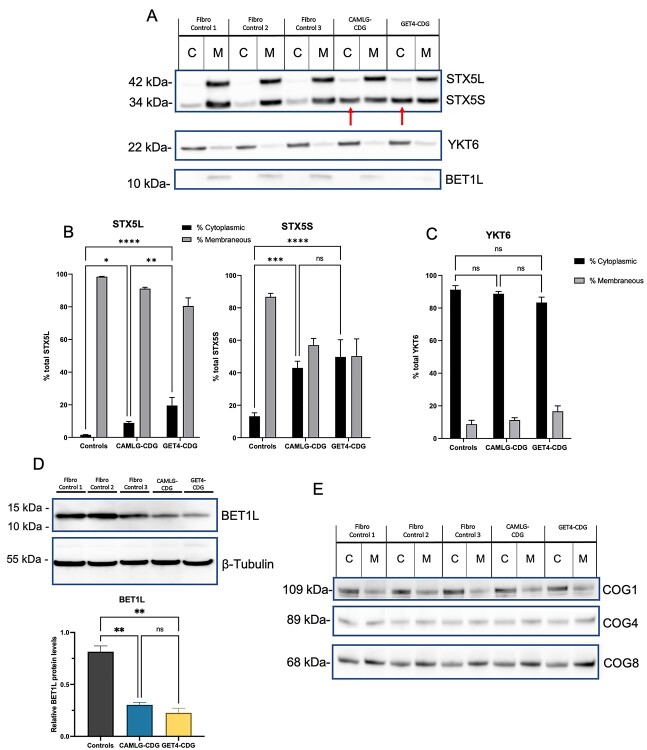
Altered localization and steady state levels of Golgi SNARE proteins in affected fibroblasts. (**A**) Crude fractionation of fibroblast lysates shows mislocalization of both the short and long forms of STX5 to the cytoplasm, the same as that identified in GET4 deficiency. Localization of the STX5-interacting Golgi SNAREs YKT6 and BET1L is normal. (**B**) The long (STX5L) and short (STX5S) forms of STX5 are significantly mislocalized to the cytoplasm of crude fibroblast fractions. (**C**) Localization of the Golgi SNARE protein YKT6 was normal in both CAMLG-CDG and GET4-CDG fibroblasts, as measured in crude fractions of lysates. (**D**) Significantly lower levels of BET1L were found in whole cell protein lysates from affected CAMLG-CDG and GET4-CDG fibroblasts. Results are the mean of three immunoblot analyses of three independently extracted cell lysates. (**E**) COG complex subunits COG1, COG4 and COG8 are normally localized in affected TRC pathway defect fibroblasts. Error bars = SEM; ^^*^^ = *P* < 0.05; ^^*^^*^^ = *P* < 0.01; ^^*^^*^^*^ ^= *P* < 0.001; ^^*^^*^^*^^*^ ^= *P* < 0.0001. C = Cytoplasmic; M = Membranous.

**Figure 6:**
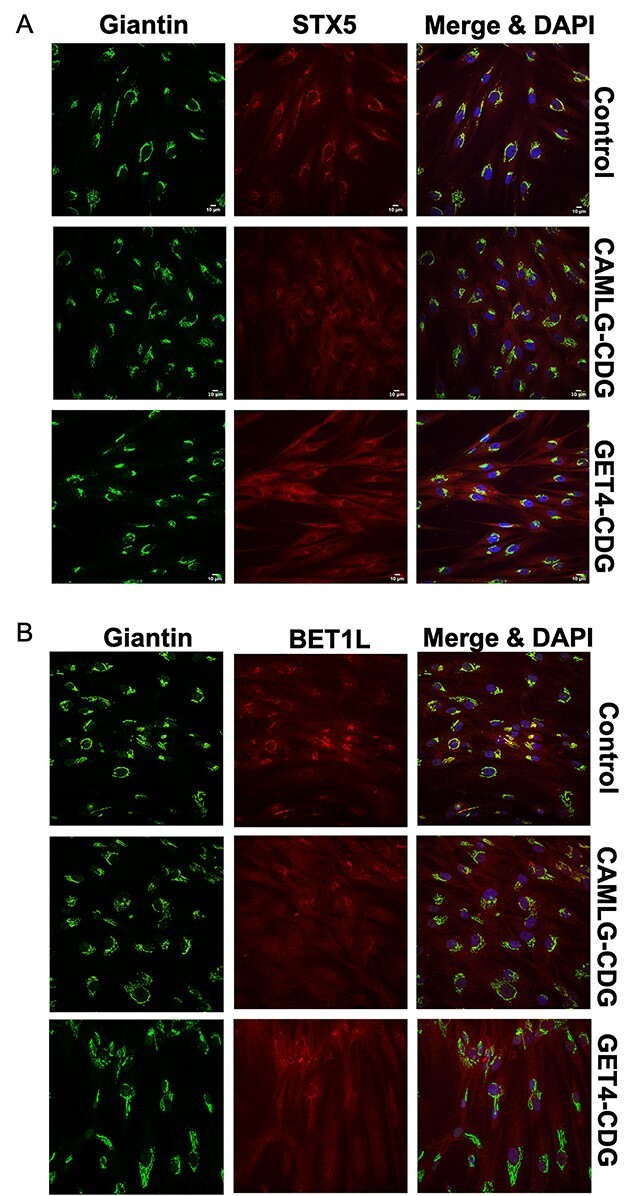
Immunofluorescence confocal microscopy confirms misregulation of Golgi SNARE proteins STX5 and BET1L in TRC pathway defects. (**A**) Syntaxin 5 is mislocalized to the cytoplasm in TRC pathway defect fibroblasts, indicated by lower colocalization with the Golgi marker giantin (see Supplementary Material, Fig. S5). (**B**) Lower levels of Golgi-localized BET1L can be seen in TRC pathway defect fibroblasts.

In order to further investigate STX5 mislocalization and that of other related SNARE proteins, the localization of YKT6 and BET1L were also measured by crude fractionation of affected fibroblasts from CAMLG and GET4-CDG fibroblasts. BET1L is also a TA protein, whereas YKT6 is not thought to be dependent on the TRC pathway for membrane insertion. Distribution of both YKT6 and BET1L between the membranous and cytoplasmic fractions was found to be normal compared to controls (Fig. 5A and C). However, total levels of BET1L in the membranous fraction appeared reduced. This was confirmed by immunoblotting from whole cell lysates, with BET1L levels at 37% and 27% of controls in CAMLG-CDG and GET4-CDG fibroblasts, respectively (Fig. 5D; *P* < 0.01). These results were also confirmed by immunofluorescence (Fig. 6B). Total YKT6 levels were normal in whole cell lysates of affected fibroblasts (data not shown).

**Figure 7:**
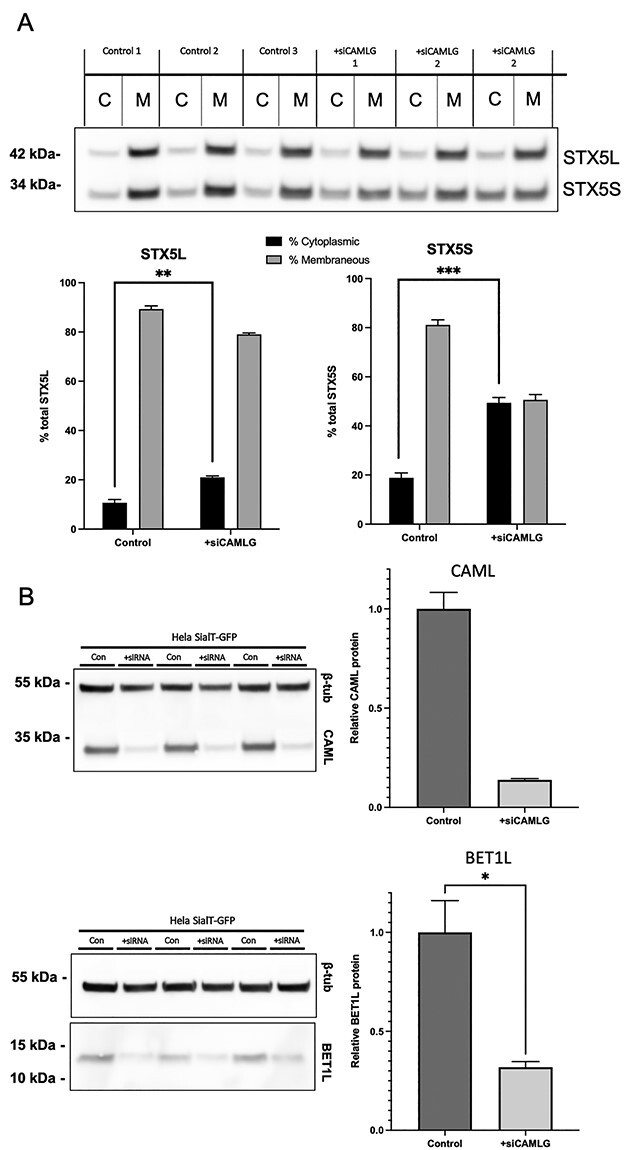
Altered localization and steady state levels of Golgi SNARE proteins STX5 and BET1L in an siRNA-mediated *CAMLG* knockdown HeLa cell line model. (**A**) Crude fractionation of fibroblast lysates shows mislocalization of both the short and long forms of STX5 to the cytoplasmic fraction upon siRNA mediated *CAMLG* knockdown. (**B**) siRNA-mediated knockdown of *CAMLG* was successful and significantly lower levels of BET1L were found in whole cell protein lysates upon *CAMLG* knockdown. Averages are a mean of three separate lysates collected from three individual transfections.^^*^^*^^*^ ^= *P* < 0.001; ^^*^^*^^ = *P* < 0.01; ^^*^^ = *P* < 0.05.

To confirm that the mislocalization of STX5 and reduced levels of BET1L in affected fibroblasts were as a specific result of TRC pathway disruption, these markers were also measured in the ST6GAL1-GFP HeLa cell line subjected to siRNA-mediated *CAMLG* knockdown. Indeed, in this model both STX5S (*P* < 0.001) and STX5L (*P* < 0.01) were significantly mislocalized to the cytoplasmic fraction from that of the membrane (Fig. 7A). Additionally, a significant (*P* < 0.01) reduction in BET1L steady-state levels was observed in whole-cell lysates, to 30.3% of levels in untreated cells (Fig. 7B). These findings therefore confirmed those identified in affected fibroblasts and the essential role of the CAML protein in STX5 localization and BET1L steady state levels.

Since several STX5-related SNARE complexes associate with the COG complex in order to facilitate vesicle fusion, the steady state as well as the subcellular localization of COG complex subunits COG1, COG4 and COG8 were also determined by crude fractionation. These were found to be normally distributed between the cytoplasmic and membraneous fractions in both CAMLG-CDG and GET4-CDG fibroblasts (Fig. 5E).

## Discussion

We report the third identified patient with a genetic disorder caused by disruption of the TRC pathway and the first caused by a pathogenic variant in *CAMLG,* encoding CAML. In addition, we confirm that this novel group of disorders lead to a CDG, in this case a combined O-linked and type II N-linked glycosylation defect characterized mostly by undersialylation, indicating late Golgi disruption. Clinical features were similar to those in one of the two other described TRC pathway disorder (GET4 deficiency; (9)) and were largely neurological, consisting of severe DD/ID, seizures, hypotonia, limb contractures and structural brain abnormalities.

CDG are a group of more than 150 genetic disorders that disrupt the glycosylation of proteins and lipids. The largest and best understood group among CDG are those that cause protein hypo-N-glycosylation. Within these there are two subgroups: CDG-I, which affects the formation or attachment of the lipid-linked oligosaccharide (LLO) to recipient nascent glycoproteins in the cytoplasm and ER, and CDG-II, which leads to aberrant downstream processing of N-glycans, typically within the Golgi apparatus. Some CDG, particularly those which disrupt Golgi trafficking such as those affecting the COG complex (14), can also lead to combined O-linked and type II N-linked glycosylation defects. Disorders affecting the TRC pathway can be placed into this group, and the three identified until now, can be termed CAMLG-CDG, GET3-CDG and GET4-CDG.

Mislocalization of STX5 was confirmed as a consistent biomarker of TRC pathway disruption in both affected fibroblasts and in HeLa cell line subjected to siRNA-mediated *CAMLG* knockdown. STX5 is important for the organization of the Golgi and for ER-Golgi transport (15) and complete loss of the short form of STX5 leads to an early lethal multisystem CDG with general N-linked and O-linked glycosylation defects (16). It is therefore likely that STX5 mislocalization plays a role in the pathomechanism of TRC pathway defects due to its role as a SNARE protein. Indeed, a large number of TA proteins targeted to the ER membrane by the TRC pathway are SNARE proteins, or interact with SNARE proteins (8).

In the case of TRC pathway dysfunction, it appears that the short form of STX5 may be affected more than the long form. This could be explained by the further observation that total protein levels of BET1L (GS15) were reduced in affected fibroblasts, and in a HeLa cell line with siRNA-mediated *CAMLG* knockdown. Perhaps, rather than, or as well as, STX5 membrane insertion being disrupted, BET1L is poorly inserted in the ER membrane and is therefore degraded prior to its inclusion in a STX5 Golgi SNARE complex. BET1L is thought to be more associated with the short form than the long form of STX5, and is required for retrograde trafficking within the Golgi (15). In fact, previous data have shown that in GET4 deficient fibroblasts, retrograde trafficking is disrupted (9). Furthermore, previous data have even shown that expression of BET1L without its transmembrane domain leads to mislocalization of STX5 (17), analogous to data presented here.

In this case of CAMLG-CDG, both the N- and O-linked glycosylation defects appear to be mainly limited to defective sialylation, with little evidence of further glycosylation abnormalities, using the markers studied. This insinuates late-Golgi dysfunction that could be difficult to reconcile with the more general defect found when STX5S is abolished, for example (16). However, this undersialylation defect does reflect that found on the knock-out of the two Golgi stacking proteins GRASP55 and GRASP65 (18). STX5 SNARE complexes are known to interact with another Golgi-stacking protein, golgin-45. When this interaction is abrogated, the integrity of the Golgi stack is reduced (19). It is possible that the glycosylation defect in cases of TRC dysfunction arrive from this relatively specific role of STX5-related SNARE proteins in Golgi stacking. It can also be speculated that the mislocalization of STX5 to the cytoplasm could in fact lead to disruption of a normal vesicle fusion process, causing an abnormal distribution of proteins within the late Golgi.

In addition, STX5 and its SNARE interactors are also known to associate with the COG complex, another well-known complex involved in CDG-II, to facilitate ER–Golgi vesicular trafficking (20). However, no obvious morphological abnormalities of the Golgi, such as those seen in COG defects, could be observed in affected CAMLG-CDG or GET4-CDG fibroblasts stained with Golgi markers GM130 or TGN46 (data not shown). We were able to show in this study that upon siRNA silencing of *CAMLG* expression in HeLa cells, a mildly abnormal Golgi morphology could be observed by the visualization of GFP-tagged ST6GAL1. It is possible therefore that in these cells, the Golgi morphology was more sensitive to disruption of the TRC pathway. This could be a cell line specific effect, or perhaps the short-term interruption using siRNA is different biochemically to that caused by disruption of the pathway by genetic variants already present in the cell line. In addition, no mislocalization of the COG complex subunits COG1, COG4 or COG8 was found in crude lysates from affected fibroblasts (Fig. 5E).

It is possible that specific glycoprotein substrates or glycosyltransferases are incorrectly trafficked within the Golgi as a downstream effect of Golgi SNARE complex dysfunction, leading to specific defective glycosylation. Additionally, perhaps morphological or other abnormalities would be more obvious in a cell-type that seems to be more affected by the pathologies related to TRC defects (i.e. neuronal, hepatic or myocytes).

It is perhaps surprising that a homozygous splice-site mutation in *CAMLG* can be compatible with life, given a mouse knockout model is embryonic lethal (21). However, it is possible that the small amount of remaining CAML protein, as measured by immunoblotting, is sufficient to provide enough insertion of TA proteins that the phenotype is attenuated. In addition, it is possible that there is some redundancy with the more recently identified SND pathway, another mechanism for the ER insertion of membrane proteins, but less well understood in mammals (22,23). This could also serve as an explanation for our own results, where several proteins that are canonically dependent upon the TRC pathway (Emerin, VAMP7, VAPB) were found to have normal subcellular localization in affected CAMLG-CDG and GET4-CDG fibroblasts (data not shown).

As well as glycosylation disorders, it is also important that CAMLG-CDG and other disorders of the TRC pathway are viewed within the context of the wider roles of TA proteins. Since TA proteins are diverse and have a multitude of roles, it is more than likely that a number of factors combine to produce the observed clinical features. Further study is needed to elucidate the precise pathomechanism.

In conclusion, three patients have been identified with pathogenic variants in TRC pathway members, each with a defect in a different member of the pathway. In two of these disorders (CAMLG-CDG and GET4-CDG), the clinical picture was remarkably similar (mostly neurological with muscular involvement, relatively severe), despite having defects in different proteins within the pathway. However, in the third disorder, GET3-CDG, the presentation was very different and extremely severe, with cardiomyopathy leading to death in infancy.

## Materials and Methods

### Genetic analysis

Next generation sequencing was performed as previously described (24).

Total RNA isolation was performed using the RNeasy Mini kit (Qiagen) including DNase treatment (Roche Diagnostics) to remove contaminating genomic DNA. Reverse transcription was then performed using 2 μg purified total RNA with the First-Strand cDNA synthesis kit (GE Healthcare).

Transcribed cDNA was used for qPCR of *CAMLG* (NM_001745.4) and *HPRT1* (NM_000194), which was used as an endogenous control for normalization. PCR primers were designed using the NCBI primer-BLAST software. Synthesis was performed by Integrated DNA Technologies. Primer sequences are found in Supplementary Material, Table S1. qPCRs were performed using the 2× LightCycler 480 SYBR Green I Master kit. Data were analyzed using the LightCycler 480 Software (Roche Applied Science).

### Biochemical analyses

CZE and transferrin IEF were performed as described (25).

### Cell culture

Primary fibroblasts from patients and controls were grown from skin biopsies. Fibroblasts were cultured in DMEM/F12 (Life Technologies) supplemented with 10% FBS (Clone III, HyClones) and the antibiotics streptomycin, penicillin and amphotericin at 37°C under 5% CO_2_. HeLa cells were cultured in the same conditions, but using standard DMEM as culture medium.

### Immunoblotting

Protein lysates were prepared by the addition of RIPA buffer (10 mm Tris–Hcl pH 7.4, 150 mm NaCl, 0.5% sodium deoxycholate, 0.1% SDS and 1× complete protease inhibitor cocktail (Sigma Aldrich)) at 4°C, mechanical lysis and incubation for 30 min followed by centrifugation at 4°C (15 000 rcf, 10 min). Total protein concentration was calculated from the supernatant using the Pierce BCA protein assay kit (Thermo Scientific). Ten micrograms of protein lysate was separated by SDS/PAGE and immunoblotted on a nitrocellulose membrane (Thermo Fisher Scientific) with respective antibodies. Signal detection was performed by autoradiography with ImageQuant LAS 4000 (GE Healthcare).quantification was performed with the ImageJ software package. All antibodies used for immunoblotting can be found in Supplementary Material, Table S2.

### Crude cell fractionation

Crude fractionate of fibroblasts and HeLa cells were carried out as previously described (9,26).

### Immunofluorescence

Cells were first grown on glass coverslips before washing once with PBS^−/−^ and incubating at room temperature for 20 min with 4% paraformaldehyde in PBS^−/−^. All subsequent steps were also performed at room temperature. The coverslips were washed three times with PBS^−/−^ for 5 min. Permeabilization was then performed by incubation with 0.2% Triton X-100 in PBS^−/−^ and then coverslips were washed three additional times with PBS^−/−^. Blocking was performed using the BlockAid™ Blocking Solution (ThermoFisher Scientific) for 60 min. Cells were then incubated for 60 min with respective primary antibodies diluted in BlockAid™, before three additional PBS^−/−^ washes and incubation with the secondary antibodies conjugated to Alexa-488 or Alexa-568 (1:500; ThermoFisher Scientific) in the dark for 60 min. The coverslips were then washed two additional times with PBS^−/−^ before incubation for 2 min with 0.2 μg/ml DAPI in PBS^−/−^ in the dark as a nuclear stain. After another wash with PBS^−/−^ and finally with water, coverslips were mounted on slides with MOWIOL 4–88 (Sigma-Aldrich) solution in glycerol as an anti-fading agent. Antibodies used can be found in Supplementary Material, Table S2. Imaging of a HeLa cell line stably expressing green fluorescent protein (GFP)-tagged β-galactoside alpha-2,6-sialyltransferase 1 (ST6GAL1) was performed as previously described (27).

Visualization of immunostained slides was performed using a Nikon C2 confocal microscope with a 40× oil immersion lens, at room temperature. Images were collected using the Nikon NIS-Elements software package and colocalization analysis performed using the ImageJ (Fiji) Coloc 2 plugin.

### Nocodazole treatment

The microtubule depolymerizing agent nocodazole (5 μm; Sigma Aldrich) was added to cultured cells for 1, 2 and 4 h before immediate fixation of cells with 4% PFA and processing for confocal microscopy as described above.

### siRNA knockdown of *CAMLG*

HeLa cells stably expressing green fluorescent protein (GFP)-tagged β-galactoside alpha-2,6-sialyltransferase 1 (ST6GAL1) were seeded in six well plates in DMEM (Lonza) + 10% FBS (Dominique Dutscher) and were immediately retrotransfected with siRNA complementary to *CAMLG* mRNA and RNAiMax (Invitrogen). For each well the following procedure was subsequently carried out: in a FACS tube, 150 μl of DMEM without serum and 9 μl of RNAiMAX are mixed. In a second FACS tube, 150 μl of DMEM and 40 pmol of siRNA are mixed. After 5 min, the first tube content is added to the second tube, and shaken. After 5 additional minutes, 300 μl of the final mix is added to each well, drop by drop. The cells are then incubated at 37°C, 5% CO_2._ After 24 h, each well is split into two wells and immediately transfected with a second round of siRNA, with only 150 μl of the mix previously described. Cells were collected 24 h after this second siRNA transfection. Desalted predesigned siRNA complementary to *CAMLG* (NM_001745) mRNA was purchased from Sigma Aldrich (Ref: SASI_Hs01_00023693).

### Lectin staining

Patients and control fibroblasts (80–90% confluence) were cultured on coverslips in six well plates in DMEM supplemented with 10% FBS, under 5% CO_2_ atmosphere. For lectin staining, coverslips were washed 3 times with PBS (+/+) (Euromedex, Souffelweyersheim, France) and mounted in a wet chamber. PNA-Cy5 or VVL-Fluorescein lectins (Vector laboratories, California, USA) were diluted at 2 μg/ml in PBS + 0.1% of BSA (Fraction V, Roche Diagnostics, Rotkreuz, Switzerland), applied on coverslips and incubated for 1 h at room temperature protected from light. The coverslips were then washed three times and fixed with 4% paraformaldehyde in PBS pH 7.3, for 30 min at room temperature. Nuclei were stained with DAPI (PBS) and coverslips were then mounted on glass slides with Mowiol.

### Glycan analysis by mass spectrometry

MALDI-TOF analysis of serum transferrin was performed as previously described (24,28).

### Statistical analysis

All analyses were carried out using GraphPad Prism version 9.20 for Mac (GraphPad Software, La Jolla, CA).

## Supplementary Material

CAMLG_supp_info_Revisions_v2_ddac055
