# Supplementary material for: CAMLG-CDG: a novel congenital disorder of glycosylation linked to defective membrane trafficking
Source: Hum Mol Genet. 2022 Mar 9;31(15):2571–81. doi: 10.1093/hmg/ddac055 (PMC9396942; doi:10.1093/hmg/ddac055)
Supplement: CAMLG_supp_info_Revisions_v2_ddac055 [file camlg_supp_info_revisions_v2_ddac055.zip › CAMLG_supp_info_Revisions_v2_ddac055.docx]

**Table S1: List of primers**

| **Target** | **Forward primer sequence (5'->3')** | **Reverse primer sequence (5'->3')** |
| --- | --- | --- |
| *CAMLG* cDNA exons 1-4 | CATCAACCGGATCATGGGCT | CCCGACAATAGAAGTGCAGC |
| qPCR – *CAMLG* | CTTTTGTTTGCAAATACTTGTCCAT | CCCGACAATAGAAGTGCAGC |
| qPCR – *HPRT1* | GCCAGACTTTGTTGGATTTG | CTCTCATCTTAGGCTTTGTATTTTG |

**Table S2: List of antibodies and lectins used for immunological analysis**

| **Antibody** | **Use** | **Species** | **Company** | **Catalogue number** |
| --- | --- | --- | --- | --- |
| Anti-CAML | Immunoblotting | Rabbit | Life technologies | PA5-89385 |
| Anti-GET4 | Immunoblotting | Rabbit | Abcam | ab93801 |
| Anti-STX5 | Immunoblotting & immunofluorescence | Mouse | Santa Cruz biotech | SC-365124 |
| Anti-BET1L | Immunoblotting & immunofluorescence | Mouse | Santa Cruz biotech | SC-135846 |
| Anti-YKT6 | Immunoblotting | Mouse | Santa Cruz biotech | SC-365732 |
| Anti-giantin | Immunofluorescence | Rabbit | Biolegend | 909701 |
| Anti-GM130 | Immunofluorescence | Mouse | BD Biosciences | 610823 |
| Anti-TGN46 | Immunofluorescence | Rabbit | Sigma Aldrich | T7576 |
| Anti-COG1 | Immunoblotting | Rabbit | - | Reynders *et al.* 2009 |
| Anti-COG4 | Immunoblotting | Rabbit | - | Reynders *et al.* 2009 |
| Anti-COG8 | Immunoblotting | Rabbit | - | Reynders *et al.* 2009 |
| Vicia villosa lectin | Lectin immunofluorescence | - | Vector Laboratories | FL-1231-2 |
| Peanut agglutinin | Lectin immunofluorescence | - | Vector Laboratories | CL-1075-1 |
| Anti-ß1-Tubulin | Immunoblotting | Mouse | Abcam | ab101019 |
| Alexa-488 | Immunofluorescence | Rabbit | ThermoFisher scientific | A-11034 |
| Alexa-568 | Immunofluorescence | Mouse | ThermoFisher scientific | A-11004 |
| Anti-Rabbit HRP | Immunoblotting | Goat | Cell signalling | 7074S |
| Anti-Mouse HRP | Immunoblotting | Goat | Cell signalling | 7076S |


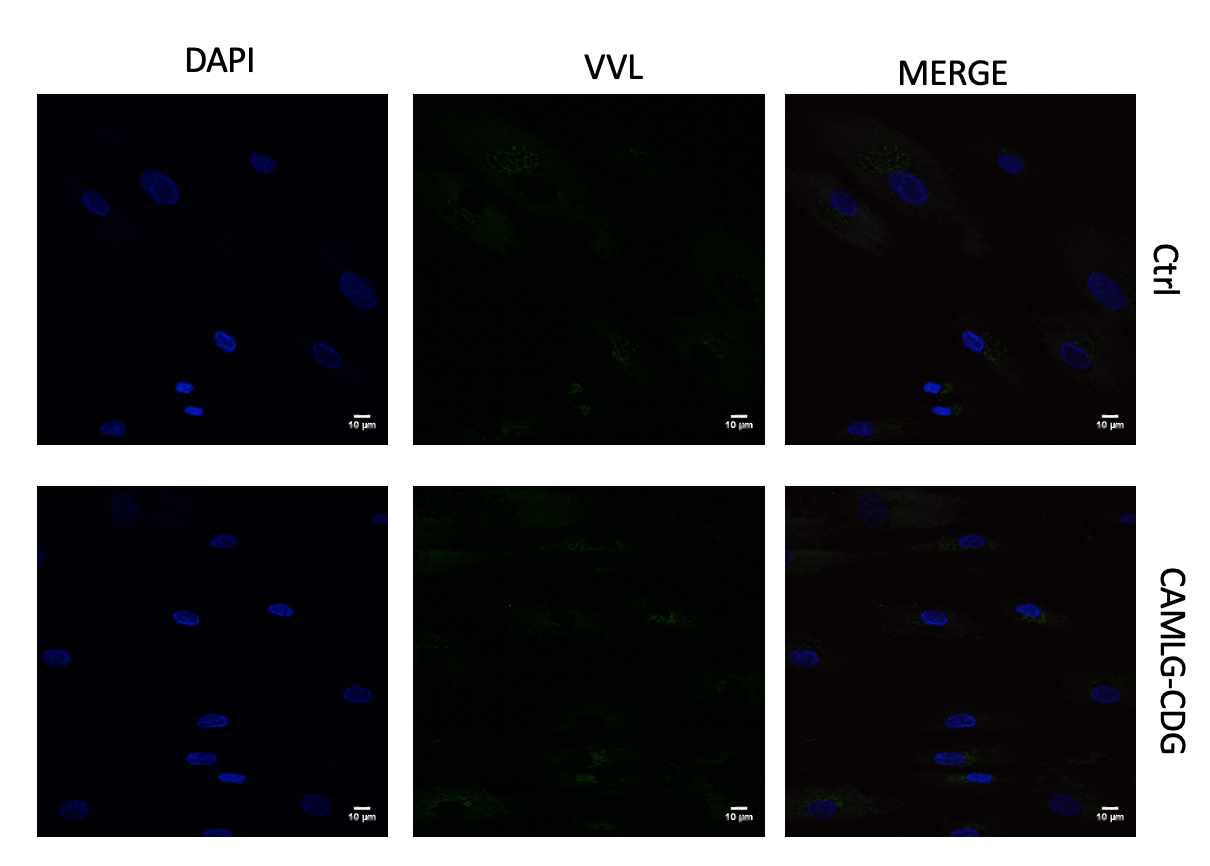


**Figure S1: Vicia villosa lectin (VVL) staining of affected CAMLG-CDG fibroblasts shows no increased signal compared to control fibroblasts.** This indicates that both GalNAc and galactose transfer onto O-glycosylation sites is normal. Images captured at 40x magnification.


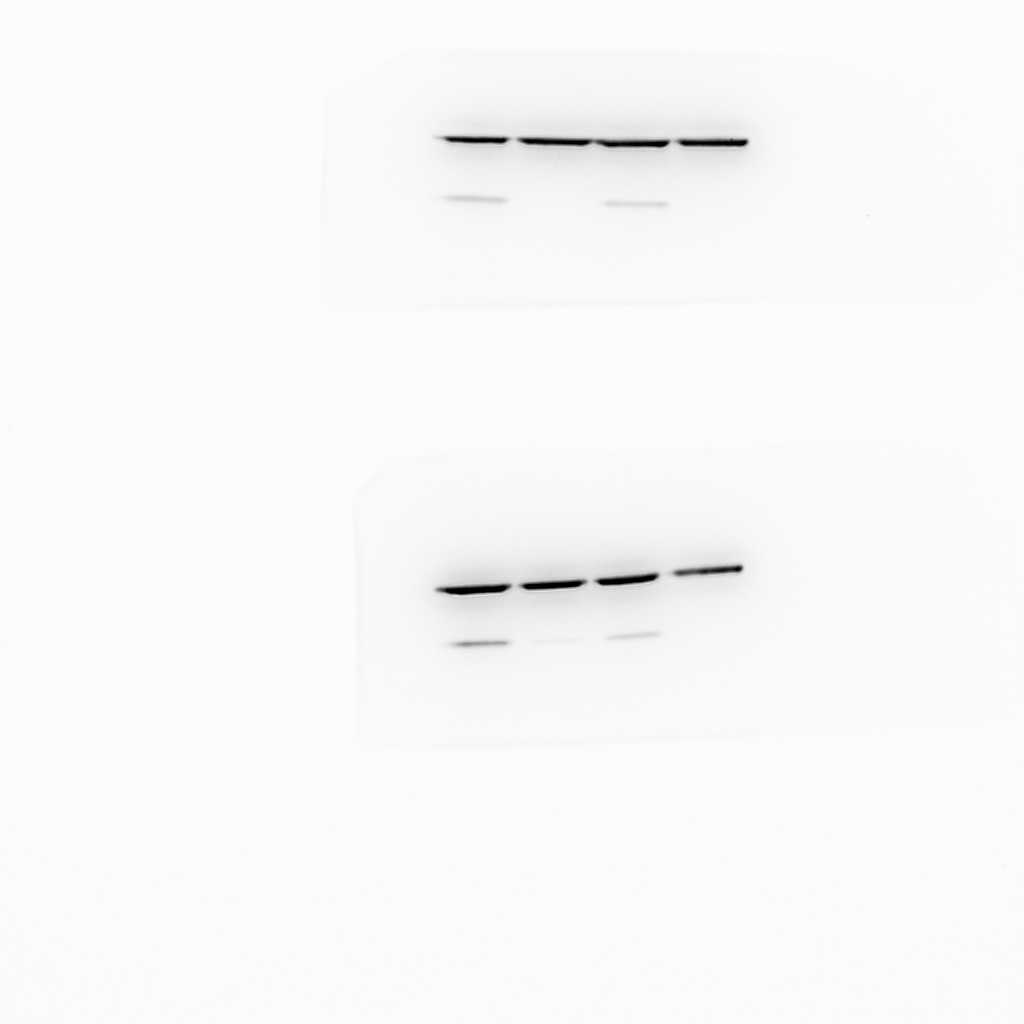

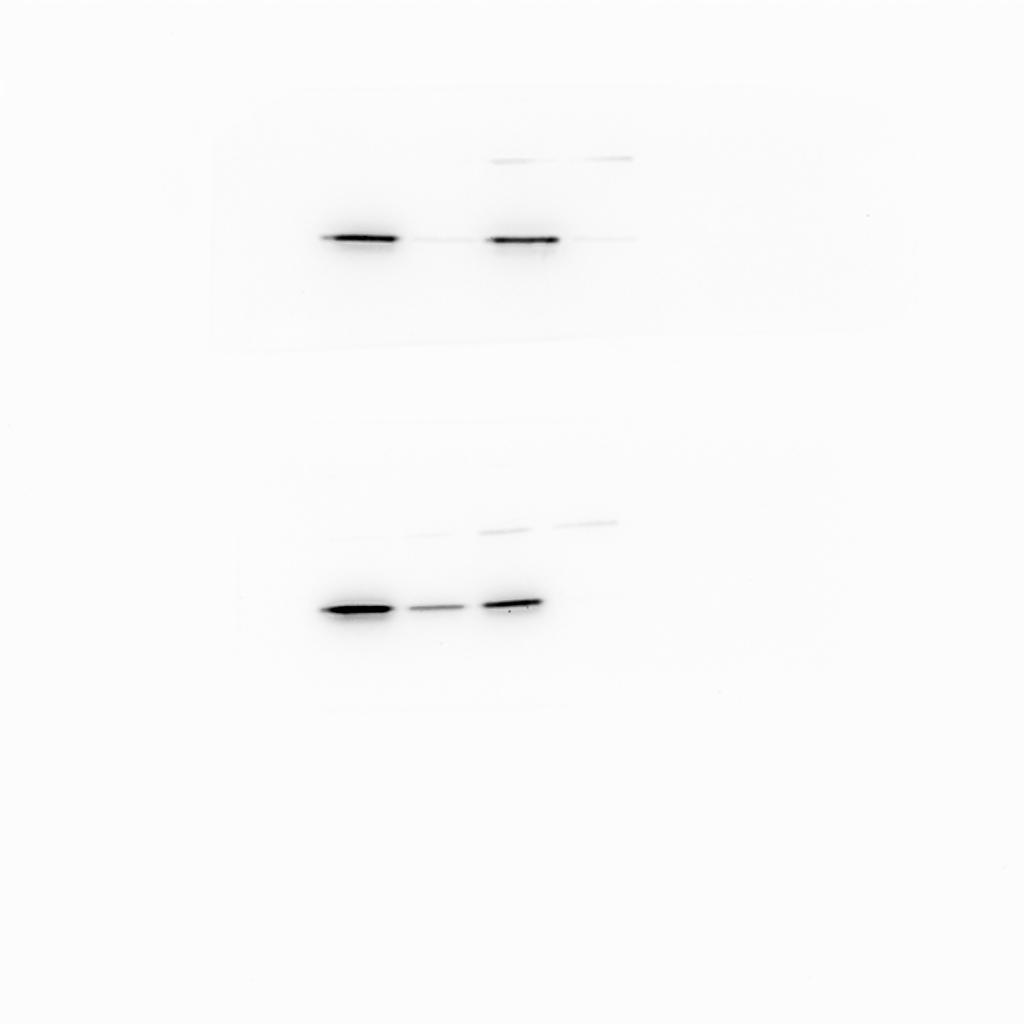


55

35

kDa

+siCAMLG

-

Hela SialT-GFP

**CAMLG**

**β-Actin**

**Figure S2: siRNA transfection of a HeLa cell line stably expressing green fluorescent protein (GFP)-tagged β-galactoside alpha-2,6-sialyltransferase 1 (ST6GAL1).** Treatment results in a reduction of CAML protein to 1.8% levels in untransfected cells, measured by normalisation to β-Actin.

**
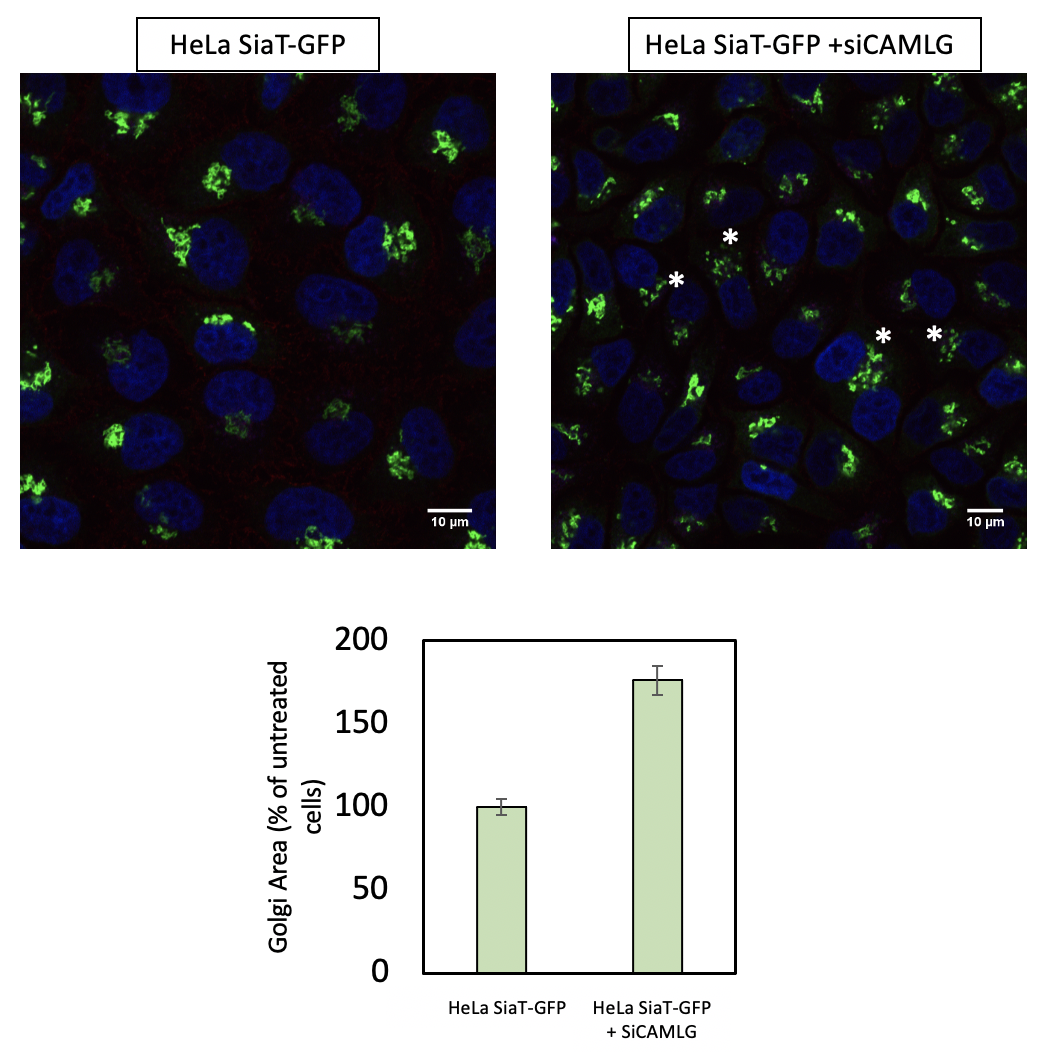
**

**Figure S3: siRNA knockdown of *CAMLG* leads to a more fragmented Golgi-related signal from stably expressed green fluorescent protein (GFP)-tagged β-galactoside alpha-2,6-sialyltransferase 1 (ST6GAL1).** Images captured at 40x magnification. * used to highlight abnormal Golgi structure.

**
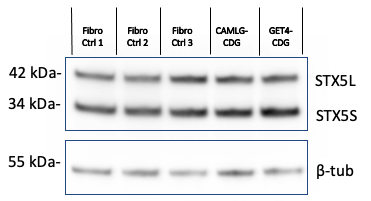
**

**Figure S4: Long and short syntaxin-5 steady state protein levels are normal in both CAML-CDG and GET4 deficient fibroblasts.**

**
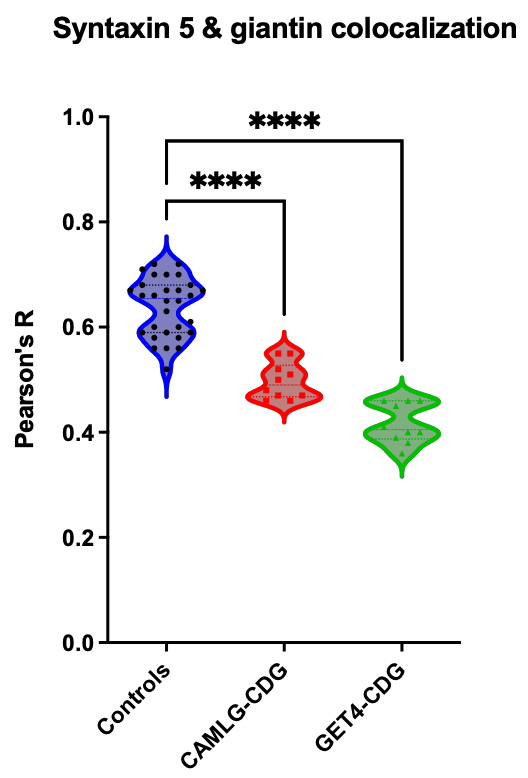
**

**Figure S5: Colocalization of STX5 and the Golgi marker Giantin is significantly reduced in CAML and GET4-deficient fibroblasts** Graph is a representation of 30 images of three separate normal fibroblast lines, 10 images of affected CAMLG-CDG fibroblasts and 10 images of affected GET4-CDG fibroblasts, 40 x magnification**.** **** = P < 0.0001.
